# The recent advances of cancer associated fibroblasts in cancer progression and therapy

**DOI:** 10.3389/fonc.2022.1008843

**Published:** 2022-09-08

**Authors:** Chenxi Wu, Jianmei Gu, Hongbing Gu, XiaoXin Zhang, Xu Zhang, Runbi Ji

**Affiliations:** ^1^ Department of Clinical Laboratory Medicine, the Affiliated People’s Hospital of Jiangsu University, Zhenjiang, China; ^2^ Jiangsu Key Laboratory of Medical Science and Laboratory Medicine, School of Medicine, Jiangsu University, Zhenjiang, China; ^3^ Department of Clinical Laboratory Medicine, Nantong Tumor Hospital, Nantong, China

**Keywords:** cancer-associated fibroblasts, tumor microenvironment, heterogeneity, tumor progression, tumor therapy

## Abstract

As an abundant component of tumor microenvironment, cancer-associated fibroblasts (CAFs) are heterogeneous cell populations that play important roles in tumor development, progression and therapeutic resistance. Multiple sources of cells can be recruited and educated to become CAFs, such as fibroblasts, mesenchymal stem cells and adipocytes, which may explain the phenotypic and functional heterogeneity of CAFs. It is widely believed that CAFs regulate tumor progression by remodeling extracellular matrix, promoting angiogenesis, and releasing soluble cytokines, making them a promising cancer therapy target. In this review, we discussed about the origin, subpopulation, and functional heterogeneity of CAFs, with particular attention to recent research advances and clinical therapeutic potential of CAFs in cancer.

## Introduction

As an important component of tumor microenvironment, CAFs are described as activated fibroblasts located in the vicinity of cancer cells without the phenotype of epithelial, cancerous, endothelial, and immune cells (1). They are elongated and spindle-shaped in morphology and have some positive markers, such as alpha-smooth muscle actin (α-SMA), fibroblast activation protein (FAP) and fibroblast specific protein 1 (FSP-1) (2). CAFs have merged as the hot-spot of cancer study; however, their phenotypic and functional heterogeneity hinders the clinical application (3). Studies have shown that CAFs could secrete a variety of chemokines, cytokines, and growth factors to facilitate tumor growth, chemotherapy resistance and immunosuppression (4). On the contrary, some studied have reported the tumor-suppressive function of CAFs in certain tumor models (5). This review summarized the heterogeneity of biological origins, phenotypic markers, and biological functions of CAFs, as well as uncovered how their heterogeneity made identification, subtypes classification and clinical therapy challenging. Our review provided a new perspective for CAF research and personalized therapy.

## The origin and transition of CAFs

Increasing evidence suggest that CAFs have different cellular origins. Though precise lineage tracing study has shown the origin of fibroblasts in healthy or injured tissues, the origins and specific activation processes of CAFs are still lacking (6, 7). Several cells may be predecessors of CAFs, such as normal fibroblasts (8), mesenchymal stem cells (MSCs) (9), pancreatic stellate cells (PSCs) (10), epithelial cells (11), endothelial cells (12), adipocytes (13), pericytes (14), hematopoietic stem cells (15) and cancer stem cells (CSCs) (16). The changes in the microenvironment where these precursor cells exist in may be a primary inducer of CAF transition (3).

As the major source of CAFs, normal fibroblasts can transform to CAFs by cytokines secreted by stromal or tumor cells. Transforming growth factor-β (TGF-β) can induce the CAF phenotype through SMAD-dependent or independent pathway (17). For example, bladder cancer cells released exosomes contain TGF-β, leading to the activation of SMAD-dependent signaling and the stimulation of normal fibroblasts to CAFs (8). Platelet-derived growth factor-D (PDGF-D) secreted by cholangiocarcinoma cells could stimulate surrounding fibroblasts to produce VEGF-C and VEGF-A, resulting in the expansion of lymphatic vasculature and tumor cell intravasation (18). In addition to cytokines, non-coding RNAs from cancer cells can also induce the conversion of resident fibroblasts to CAFs. Exosomes derived from hepatocellular carcinoma cells were rich in miR-1247-3p, which activated β1-integrin-NF-κB signaling through targeting B4GALT3 in fibroblasts (19). In lung adenocarcinoma, miR-200 deficiency in cancer cells promoted the expression of Jagged1/2 and the activation of Notch in adjacent CAFs, which reprogrammed CAFs from a quiescent state into an active pro-tumorigenic state (20). Additionally, the hypoxia microenvironment also contributes to the activation of resident fibroblasts. Hypoxia was related to the accumulation of ROS, the activation of the HIF-1α signaling pathway in hepatocellular carcinoma cells, and the enhanced expression of FAP in surrounding fibroblasts (21).

MSCs are another important source of CAFs. The transformational potential of MSCs into CAFs was first proved in breast cancer (9). TGF-β secreted by cancer cells recruited MSCs and maintained the differentiation of MSCs into CAFs (22). In colorectal cancer, the high level of stromal cell-derived factor-1 (SDF-1) upregulated the expression of chemokine receptor 4 (CXCR4) and TGF-β in MSCs, leading to the transformation of MSCs (23). In epithelial ovarian cancer, the elevated expression of STAT4 in epithelial cells induced MSCs derived from adipose and bone marrow to obtain CAF-like features, which in turn promoted EMT and peritoneal metastasis of ovarian cancer by secreting CXCL12, IL-6 and VEGF-A (24). In addition to the stimulation of cancer cells, changes in tumor microenvironment like pH can also stimulate the transformation of MSCs. PH induced activation of MSCs to CAFs was decreased by upregulating the expression of proton-sensing G-protein-coupled receptor68 (GPCR68) and activating downstream effector-Yes-associated protein (YAP) in MSCs (25).

The other cellular origins of CAFs have been reported. For example, PSCs could transform to CAFs in pancreatic cancer (10). In pancreatic ductal adenocarcinoma, the IL-1 signaling cascade led to JAK/STAT activation and induced an inflammatory CAF state (26). Epithelial or endothelial cells are found to be the probable origins of CAFs through epithelial-to-mesenchymal transition (EMT) or endothelial-to-mesenchymal transition (EndMT). The human nasal epithelial cells were activated and displayed CAF phenotypes such as FSP or FAP through EMT when they were exposed to matrix metalloproteinase (MMP)-9 (11). TGF-β could induce proliferating endothelial cells into fibroblast-like cells (12). In addition, a recent study reported that tumor cells induced adipocytes to CAFs by activating Wnt/β-catenin signaling in ovarian cancer (13). Cancer cells, especially cancer stem cells, have also been demonstrated to be a source of CAFs through the action of TGF-β (15). Besides these sources of CAFs mentioned above, there also exist some uncommon origins, such as pericytes, hematopoietic stem cells, which needs further exploration (14, 16).

**Figure 1:**
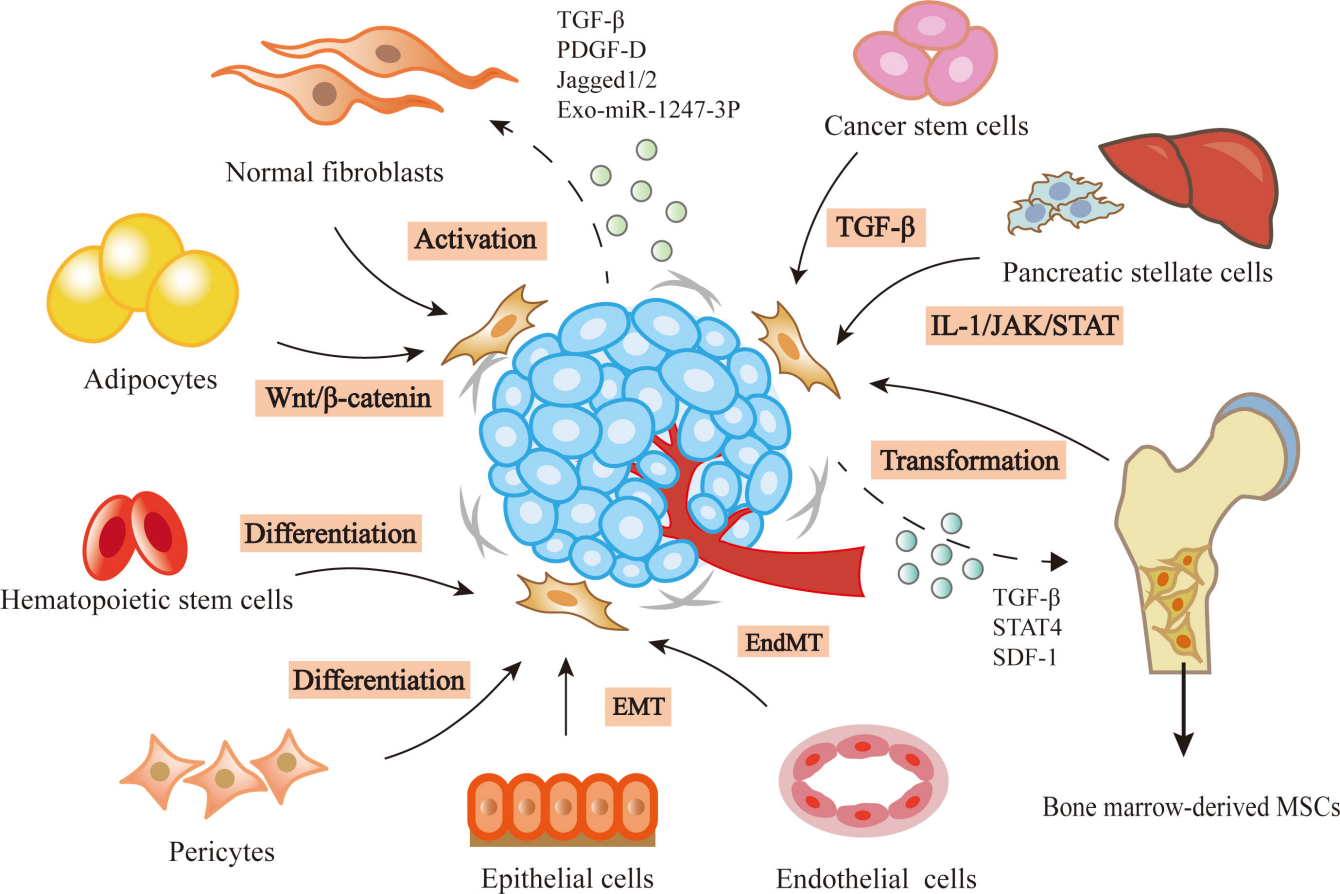
Heterogenous origins of CAFs. In the tumor microenvironment, lots of precursor cells can be transformed into CAFs by the stimulation of cancer cells, such as normal fibroblasts, bone marrow-derived MSCs, pancreatic stellate cells, epithelial cells, endothelial cells, adipocytes, caner stem cells, hematopoietic stem cells and pericytes.

In brief, the activation of CAFs is mainly regulated by different cytokines and signaling pathways of cancer niche (**Figure 1**). Although the origins of CAFs in solid tumors were not fully elucidated, using lineage tracing technologies to track CAF transition may provide a solution in the future.

## Phenotypic identification and subtype classification of CAFs

The altered protein profiles can be used to identify or isolate CAFs. According to the distinct phenotypic markers, CAFs can be divided into several subpopulations and some of them partially overlap. In this part, we will present the phenotypic differences and subtype classification of CAFs, and provide some suggestions for identifying different CAF populations.

There are several typical CAF markers, such as FAP, α-SMA, FSP-1, PDGFR-α, PDGFR-β, and Thy-1 (27). Despite the diversity of biomarkers, the isolation of CAFs from cells remains a challenge due to low specificity. For example, α-SMA and FAP were highly presented in pericytes, lymphatic endothelial cells and fibroblast reticular cells. Similarly, vimentin was present in endothelial cells, smooth cells and tumor cells (28). Additionally, with the continuous optimization of detection technology, the researchers identified uncommon PSC-derived CAF subsets in pancreatic ductal adenocarcinoma tissues. These CAFs located away from cancer cells, lacked elevated α-SMA expression, and secreted IL-6 and other inflammatory mediators (10). The results highlighted the importance of considering multiple indicators in CAF identification. In addition to classical phenotypic markers, some new ones are studied in recent years. In pancreatic cancer, the high expression of caveolin-1 (Cav-1) in CAFs was associated with the invasiveness of cancer cells and poor prognosis of patients (29). The same results were further proved in lung adenocarcinoma (30). Similarly, a recent study reported that the melanoma cell adhesion molecule+ (MCAM+) CAFs induced by TGF-β in colorectal cancer patients were associated with poor prognosis (31). Another study concluded that focal adhesion kinase (FAK) activity in CAFs was increased in PDAC tissues compared with healthy ones and the FAK+ CAFs could be an independent prognostic marker (32).

Based on surface markers, CAFs are classified into different subtypes that display distinctive secretory phenotypes and perform specific biological functions in dynamic tumor environment, as summarized in **Table 1** (33). In a mouse model of pancreatic ductal carcinoma, the researchers demonstrated the existence of myofibroblastic CAFs (myCAFs), inflammatory CAFs (iCAFs) and antigen-presenting CAFs (apCAFs) by single-cell RNA sequencing. MyCAFs were characterized by the expression of α-SMA, TAGLN, MYL9, TPM1, TPM2, MMP11, POSTN and HOPX, which could promote the proliferation, invasion and metastasis of tumor cells. ICAFs could promote metastasis and angiogenesis by producing inflammatory cytokines and chemokines such as IL-6, IL-8, CXCL1, CXCL2, CCL2, CXCL12 and Ly6c. ApCAFs had immunomodulatory capacity in pancreatic ductal adenocarcinoma. They expressed MHC II, Saa3, Slp and could activate CD4+ T cells in an antigen-specific manner in the model system (34). Another study reported four CAF subtypes in pancreatic ductal adenocarcinoma based on transcriptomic analysis. These four subgroups, named A-D, could be distinguished by differential expression of three markers, periostin (POSTN), myosin‐11 (MYH11) and podoplanin (PDPN). Patients with the dominant subtype-C had prolonged survival, whereas those with the dominant subtype D had the worst prognosis, suggesting that specific tumor-stromal interactions are associated with adverse outcomes (35). Furthermore, a novel subtype of CAFs with a highly activated metabolic state (meCAFs) was identified in PDAC. MeCAFs had highly activated glycolysis, and patients with abundant meCAFs had a higher risk of metastasis and poor prognosis, but showed a dramatically better response to immunotherapy (36).

**Table 1:** CAF subtypes and their markers.

CAF subtypes	Phenotypic markers	Functions	Detecting techniques	Cancer types	Refs
myCAF (myofibroblastic CAF) iCAF (inflammatory CAF) apCAF (antigen-presenting CAF)	α-SMA, TAGLN, MYL9, TPM1, TPM2, MMP11, POSTN, HOPX IL6, IL8, CXCL1, CXCL2, CCL2, CXCL12, Ly6c MHC II, Saa3, Slpi	Promoting proliferation, invasion and metastasis Promoting metastasis and angiogenesis Activating CD4+ T cells	Single-cell RNA sequence	Pancreatic ductal carcinoma (mouse)	(34)
CAF-A CAF-B CAF-C CAF-D	POSTN POSTN, MYH11, PDPN PDPN Not determined	Associated with intermediate prognosis Associated with intermediate prognosis Associated with better prognosis Associated with poorer prognosis	Single-cell RNA sequence	Pancreatic ductal carcinoma (human)	(35)
meCAF (Metabolic state CAF)	CD74 and HLA-DRA	Promoting metastasis	Single-cell RNA sequence	Pancreatic ductal carcinoma (human)	(36)
CAF-S1 CAF-S2 CAF-S3 CAF-S4	FAP^High^, CD29^Med-High^, αSMA^High^, PDPN^High^, PDGFRβ^High^ FAP^Neg^, CD29^Low^, αSMA^Neg-Low^, PDPN^Low^, PDGFRβ^Low^ FAP^Neg-Low^, CD29^Med^, αSMA^Neg-Low^, PDPN^Low^, PDGFRβ^Low-Med^ FAP^Low-Med^, CD29^High^, αSMA^High^, PDPN^Low^, PDGFRβ^Med^	Mediating EMT Making up of healthy tissues Making up of healthy tissues Inducing cancer invasion	Flow cytometry, immunohistochemistry and RNA-sequencing	Breast cancer (human)	(37)
CD10+ GPR77+ CAF	CD10, GPR77	Promoting tumor formation and chemoresistance	Single-cell RNA sequence	Breast and lung cancer (human)	(38)
vCAF (vascular CAF) mCAF (matrix CAF) cCAF (cycling CAF) dCAF (developmental CAF)	Desmin Fibulin-1, PDGFR-α Similar with vCAF Scrg1	Invading tumor stroma Regulating tumor immune response Similar with vCAF Promoting tumor formation	Single-cell RNA sequence	Breast cancer (human)	(39)
CAF-C1 CAF-C2	BMP4 α-SMA	Modulating cancer cells proliferation and stemness Inhibiting cancer proliferation	Single-cell RNA sequence	Oral carcinoma (human)	(40)
eCAF (extracellular matrix CAF)	POSTN	Promoting cancer invasion	Single-cell RNA sequence	Gastric cancer (human)	(41)
CAF-A CAF-B	MMP2, DCN, COL1A2 ACTA2, TAGLN, PDGFA	Remodeling extracellular matrix Expressing cytoskeletal genes	Reference component analysis(RCA)	Colorectal cancer (human)	(42)
Subtype l Subtype II Subtype III	HGF, FGF7 FGF7 Low HGF and FGF7	Broad tumor promotion Modest tumor promotion Minimal tumor promotion	Single-cell RNA sequence	Non-small lung cancer (human)	(43)
Activated myofibroblast Phenotype Mesenchymal stromal cell phenotype	α-SMA, vimentin, FAP, collagen 1α, PDGFRα CD90, CD73, CD105, CD29, CD44, CD166	Enhancing the stemness of cancer cells Regulating immunosuppression	Flow cytometry	Hepatocellular carcinoma (human)	(44)
FAP-high CAF FAP-low CAF	FAP, TGF-β, IL-6, COL11A1, SULF1, CXCL12 DLK1, COLEC11, TCF21	Regulating cancer invasion and immune regulation Regulating glucose homeostasis and lipid metabolism	Quantitative RT-PCR	High-grade serous ovarian cancer (human)	(45)

In human breast cancer, four CAF subgroups, known as S1-S4, have been identified by flow cytometry, immunohistochemistry, and RNA sequencing. They can be distinguished according to the expression of FAP, CD29, αSMA, PDPN and PDGFRβ. CAF-S1 stimulated cancer cell migration and mediated EMT transition through the activation of CXCL12 and TGF-β. CAF-S4 induced cancer invasion through NOTCH signaling. The study also found that patients with high levels of CAF-S4 in lymph nodes were prone to late distant metastases, which could be a potential prognostic marker for breast cancer (37). Furthermore, two new cell surface molecules, CD10 and GPR77, can define a CAF subset associated with chemoresistance and low survival in patients with breast cancer and lung cancer. CD10+ GPR77+ CAFs accelerated cancer progression by providing a survival niche for cancer stem cells, and the functional CAF subset could be specifically recognized and isolated, suggesting an effective therapeutic strategy for CSC-driven solid tumors (38). Bartoschek and colleagues defined four spatially and functionally distinct CAF subpopulations through single-cell RNA sequencing in breast cancer. According to different functions, these subgroups were named as vascular CAFs (vCAFs), matrix CAFs (mCAFs), cycling CAFs (cCAFs) and developmental CAFs (dCAFs). VCAFs originated from perivascular location, expressed genes controlling angiogenesis, and invaded tumor stroma during tumor progression. MCAFs were offspring of resident fibroblasts and regulated the tumor immune response. CCAFs were proliferative fragment of vCAFs and had different expression of cell cycle genes. DCAFs underwent EMT and shared expression patterns with tumor epithelium. Thus, the phenotypic and functional heterogeneity of CAFs can be attributed to their different origins (39).

In oral carcinoma, CAFs were grouped into two distinct clusters based on the expression difference of α-SMA. CAF-C1 had low α-SMA-scores and was more supportive for cell proliferation but suppressive for the growth of stem-like cancer cells (SLCCs). BMP4 played a determinant role in C1-type CAF-mediated suppression of SLCCs. However, CAF-C2 had the opposite effects on tumor cells (40). In gastric cancer, the researchers identified a new CAF subset defined as extracellular matrix CAFs (eCAFs). The subset had high expression of POSTN, which could support the adhesion and migration of epithelial cells, as well as be a prognostic marker for gastric cancer (41). In colorectal cancer, two distinct CAF subtypes, named CAF-A and CAF-B, were identified depending on their differential expressions. CAF-A expressed markers related to extracellular matrix remodeling, such as Matrix metalloproteinase-2 (MMP2), decorin (DCN) and collagen 1A2 (COL1A2). CAF-B cells expressed markers of myofibroblasts such as actin alpha 2 (ACTA2), transgelin (TAGLN) and platelet-derived growth factor A (PDGFA) (42). Hu and colleagues identified three subtypes of CAFs in non-small lung cancer. Subtype I highly expressed hepatocyte growth factor (HGF) and fibroblast growth factor 7 (FGF7), and had strong protective effects against cancer. Subtype II expressed FGF7 and had moderate protection against cancer. Subtype III had minimal protection (43). In hepatocellular carcinoma, CAFs isolated from fresh tumor tissues could be divided into activated myofibroblast phenotype and a mesenchymal stromal cell phenotype. They could enhance the stemness of cancer cells and modulate immunosuppression, respectively (44). In high-grade serous ovarian cancer, the CD49e+ CAF population was divided into two subgroups, FAP-high and FAP-low group. The FAP-high subgroup could regulate cancer invasion and immunomodulation, whereas the FAP-low group could regulate glucose homeostasis and lipid metabolism (45).

In summary, CAFs can be divided into several specific subpopulations in different tumor models based on surface markers and protein profiles (**Table 1**). These studies suggest that CAFs are a cell state rather than end-point of differentiation. Because the subtypes are dynamic, they can be mutually transformed under the influence of cancer status and drug treatment. For example, when CAFs are isolated from cancer tissues and cultured *in vitro*, CAF subpopulations may change their phenotype. Furthermore, the transition could also occur in different tumor types, even in different parts of the same tissue, so more advanced detection techniques and strategies are needed to further identification. Single-cell RNA sequencing is a cutting-edge technology that can investigate the transcriptome and related markers of individual cells, which may help to more accurately classify CAF subtypes in further studies (46). Additionally, new technologies such as mass spectrometry-based time-of-flight flow cytometry (CyTOF) (47), multiple flow cytometry (48) and multiple immunostaining (49) are helpful to identify CAF subtypes. During the detection of CAF subtypes, it is necessary to guarantee the number of patients to ensure the production of several cell subsets. Second, fresh samples are crucial in the current single-cell RNA sequencing strategy. Finally, batch effects may be involved between batch loaded samples (50). In addition to the inclusion of more molecular markers, the different functions, different positions in cancer tissues, and even different tumor stages of CAFs should also be considered to achieve a more detailed classification of CAFs.

## Functional heterogeneity of CAFs in cancer biology

### CAFs promote tumorigenesis and metastasis

CAFs play a dynamic role in proliferation, invasion and metastasis of tumors, and its mechanism is gradually elucidated. In lung adenocarcinoma, CAFs secreted SDF-1 to promote the expression of CXCR4, β-catenin and peroxisome proliferator activated receptor δ (PPARδ) in tumor cells, and enhance cancer invasiveness and EMT (51). In breast cancer, CAFs secreted IL-32 to induce an interaction between integrin β3 and the RGD motif, activate p38 MAPK in tumor cells, leading to increased expression of EMT markers (52). TGF-β and inflammatory cytokines secreted by breast cancer cells induced CAFs to express gremlin 1 (GREM1), abrogating BMP/SMAD signaling and promoting stemness and invasion of cancer cells (53). In gastric cancer, downregulation of miR-214 in CAFs resulted in a high expression of Fibroblast Growth Factor 9 (FGF9), promoting EMT and tumor metastasis (54). In human colorectal cancer, CAFs promoted cancer proliferation, EMT and metastasis by secreting pro-inflammatory factors, such as IL-6, IL-8 and exosomal miRNA-92a-3p to activate Wnt/β-catenin pathway as well as inhibit mitochondrial apoptosis (55, 56).

### CAFs induce chemoresistance

Tumor matrix is not only the material support but also an important regulator of cancer cells. They create a complex signaling network to promote drug resistance in tumor cells after drug treatment (57). In patients with breast and lung cancer, phosphorylation and acetylation of p65 activated NF-κB to produce CD10+GPR77+ CAFs. They provided a survival niche for cancer stem cells to achieve tumor formation and chemoresistance (37). Similarly, IL-11 secreted by CAFs induced STAT3 phosphorylation and increased the expression of anti-apoptotic proteins Bcl-2 and Survivin in lung adenocarcinoma. These protected cancer cells from cisplatin-induced apoptosis, thereby promoting chemoresistance (58). Exosomes derived from CD63+ CAFs contained miR-22 and mediated tamoxifen resistance in breast cancer by targeting ERα and PTEN (59). In gastric cancer, the USP7/hnRNPA1 axis was activated and miR-522 was expressed in CAFs after cisplatin and paclitaxel treatment, leading to ALOX15 inhibition and reduced lipid-ROS accumulation in cancer cells, ultimately resulting in decreased chemosensitivity (60). CAFs could also secrete IL-8 and activate the NF-κB signaling pathway in gastric cancer to mediate chemoresistance (61). In pancreatic ductal carcinoma, CAFs secreted SDF-1 to upregulate the expression of SATB-1 in cancer cells and mediate gemcitabine resistance (62). Similarly, CAFs promoted pancreatic cell proliferation and drug resistance by releasing exosomes containing the chemoresistance inducing factor, Snail (63).

### CAFs mediate immunosuppression

CAFs can promote the immunosuppression of cancer cells by secreting TGF-β, IL-6, CXCL12 and CCL2, thereby preventing cytotoxic T cell activity and recruiting immunosuppressive populations (64). There was a significant increase in regulatory T cells (Tregs) in paracancerous tissues, which secreted TGF-β and IL-10 to inhibit the activation of tumor-site effector T cells. In breast cancer, CAF-S1 enhanced the ability of Tregs to suppress T effector proliferation, and then promoted immunosuppressive (65). A new subset of CAFs that expressed CD68 was found in esophageal squamous cell carcinoma. The recurrence rate of patients with low-CD68 CAFs was higher. Knockdown of CD68 in CAFs upregulated the secretion of CCL17 and CCL22 by tumor cells to enhance Treg recruitment (66). MiR-92-containing exosomes from CAFs induced the expression of programmed cell death receptor ligand 1 (PD-L1) in breast cancer and raised the apoptosis of T cells (67). Similarly, in melanoma and colorectal cancer cells, CAFs led to the high expression of PD-L1 and the activation of PI3K/AKT signaling, resulting in the disappearance of T cells in the anti-tumor immune response (68). Furthermore, CAFs could inhibit an anti-tumor immune response by inhibiting dendritic cells which are necessary for T lymphocytes activation. In a recent study, CAFs secreted WNT2 in esophageal squamous cell carcinoma and colorectal cancer. WNT2 suppressed the dendritic cells to act on the anti-tumor T cell response *via* SOCS3/p-JAK2/p-STAT3 signaling (69). Additionally, CAFs could also reduce immune efficiency by recruiting granulocytes and monocytes, and suppressing dendritic cell functions (70, 71). For example, increased expression of IL-33 in metastases-associated fibroblasts stimulated type 2 immunity and mediated the recruitment of eosinophils, neutrophils and inflammatory monocytes, influencing the function of these immune cells in tumor tissues (72).

### CAFs exert tumor suppression effect

**Figure 2:**
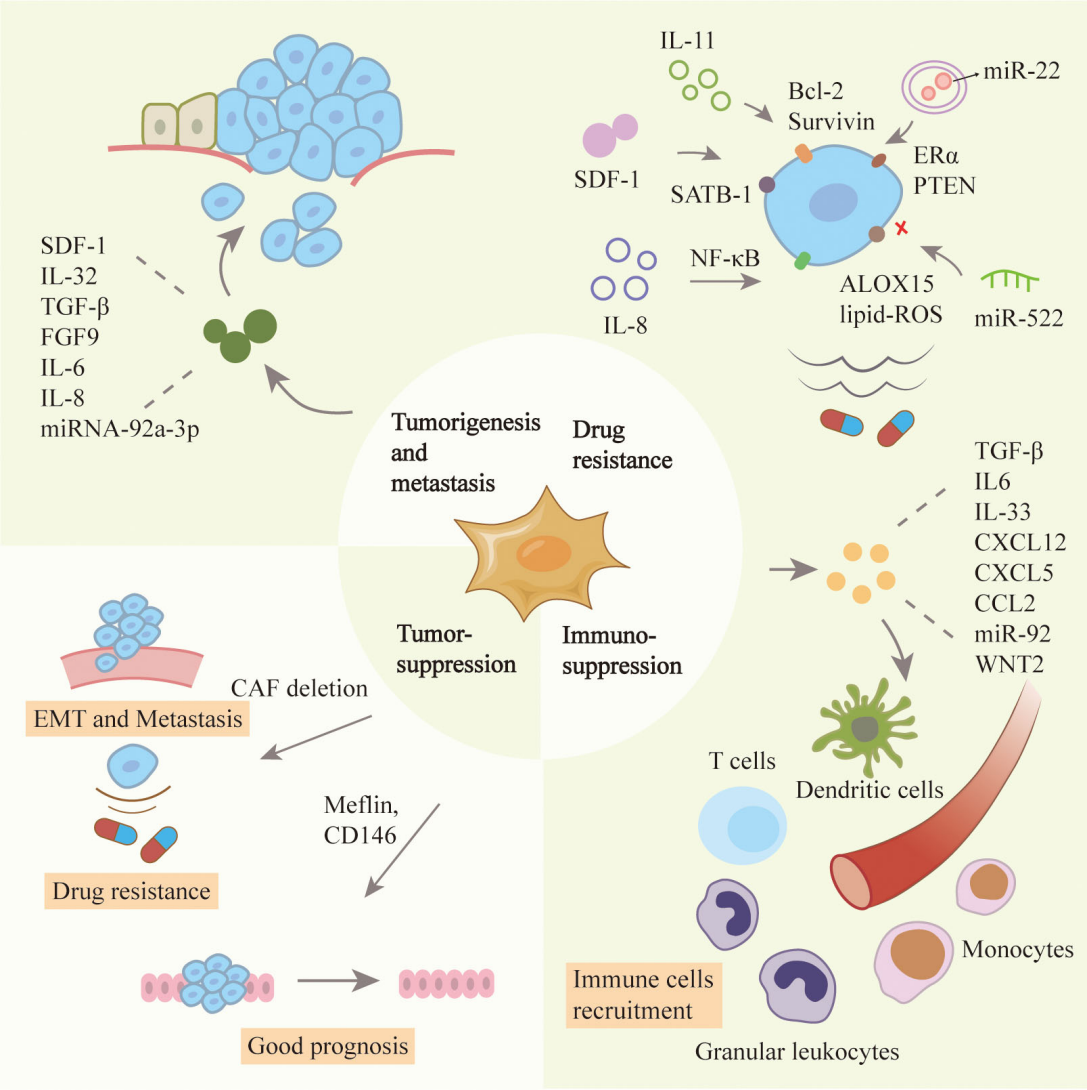
Roles of CAFs in tumor progression. CAFs have heterogenous functions in the tumor microenvironment including tumor promotion and suppression ones. CAFs can stimulate the proliferation, metastasis and drug resistance of cancer cells, and inhibit the effect of immune cells. CAFs have also been reported to inhibit tumors because their absence can affect the prognosis of patients.

Although the studies mentioned above have revealed the cancer-promoting function of CAFs, some studies have also reported the tumor suppression effects of CAFs. In a mouse model of pancreatic ductal carcinoma, ablation of CAFs was first proven to be associated with worse tumor progression, further supporting the concept of CAFs heterogeneity in the tumor microenvironment (73). In mice with pancreatic cancer, the absence of α-SMA+ myofibroblasts led to hypoxia enhanced and EMT turnover. In patients with pancreatic ductal carcinoma, fewer myofibroblasts were related to increased drug resistance and reduced survival. Another study reported that deletion of sonic hedgehog (SHH) decreased the formation of fibroblast-rich desmoplastic stroma, increased vascularity and enhanced tumor proliferation (74). In estrogen receptor-positive (ER+) breast cancer, CD146+ CAFs could maintain ER expression, estrogen-dependent proliferation and tamoxifen sensitivity (75). Furthermore, a recent study reported the presence of two populations of CAFs with different functions, namely, cancer-promoting and cancer-restraining. Meflin, a marker of mesenchymal stromal cells to maintain their undifferentiated state, was expressed on pancreatic stellate cells in pancreatic ductal carcinoma. The results of situhybridization analysis of 71 human pancreatic ductal carcinoma tissues showed that the infiltration of Meflin-positive CAFs was related to good prognosis. In a mouse model of pancreatic ductal carcinoma, Meflin deficiency led to significant tumor progression in poorly differentiated histology (76). The functional heterogeneity of CAFs in certain cancer types was highlighted in **Figure 2**.

## Treatment strategies for CAFs

**Figure 3:**
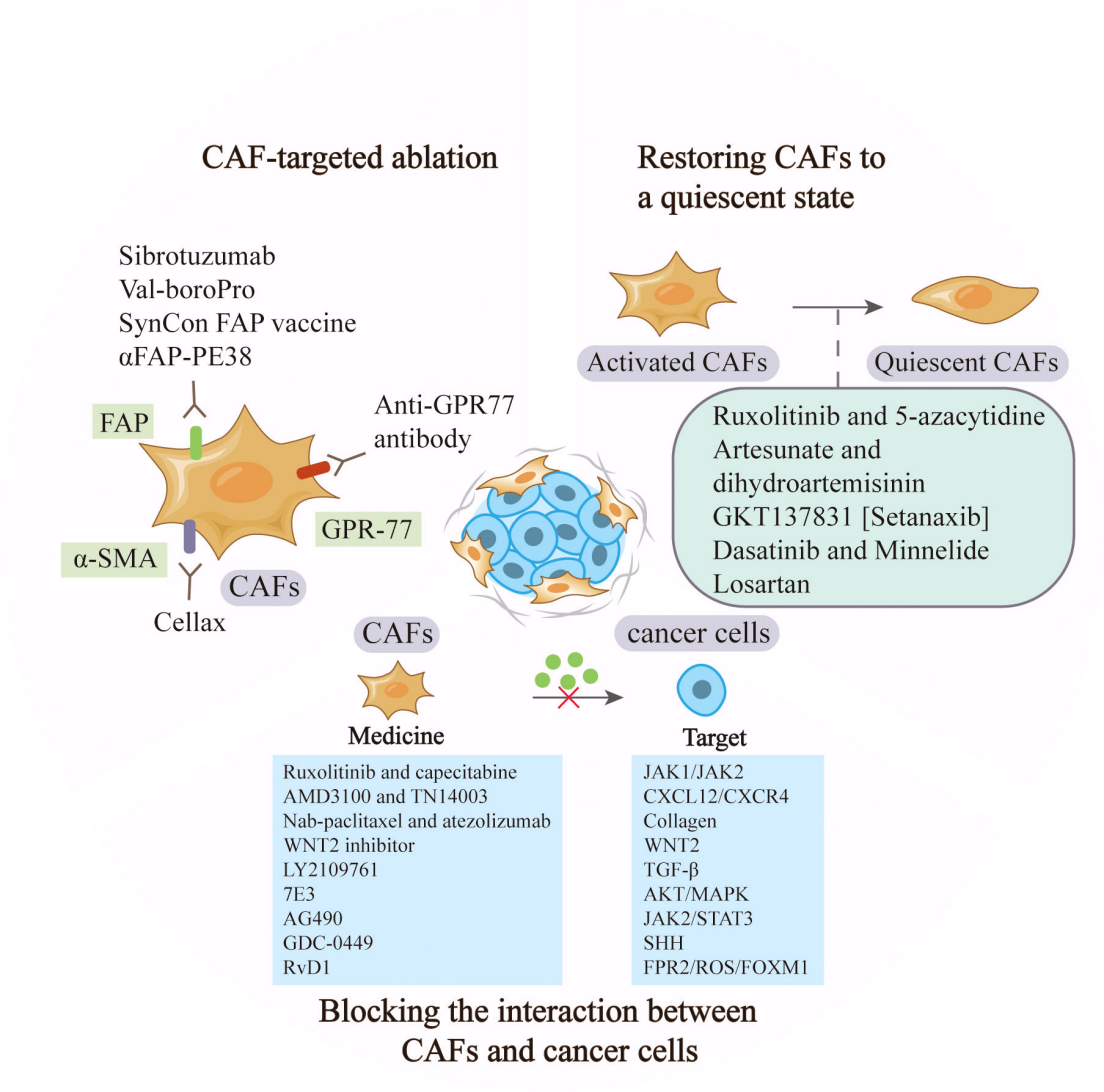
Anti-cancer strategies based on CAFs. CAF-based therapy can be achieved by targeting the markers to ablate CAFs, restoring activated CAFs to quiescent ones, and blocking the signaling between CAFs and tumor cells such as JAK1/JAK2 and CXCL12/CXCR.

CAFs play a vital role in cancer occurrence and development by regulating the proliferation, invasion and chemoresistance of tumor cells. The abundance in tumor microenvironment and the diverse tumor-supportive roles of CAFs make them an ideal therapeutic target (77). The recent advances in cancer therapy by targeting CAFs were summarized in **Table 2** and **Figure 3**.

**Table 2:** Treatment strategies based on CAFs.

Drugs	Mechanism	Cancer models	Biological effects	State	Refs
**CAF-targeted ablation**					
Sibrotuzumab	Deplete FAP+ CAFs	Colorectal cancer and non-small cell lung cancer	Inhibit tumor growth	Phase l	(78)
Val-boroPro	Deplete FAP+ CAFs	Colorectal cancer	Inhibit tumor growth	Phase II	(79)
SynCon FAP DNA vaccine	Deplete FAP+ CAFs	Lung, prostate, breast cancer	Enhance immune response	Preclinical	(80)
αFAP-PE38	Deplete FAP+ CAFs	Breast cancer	Inhibit tumor growth	Preclinical	(81)
Cellax	Deplete αSMA+ CAFs	Breast cancer	Deplete tumor stroma	Preclinical	(82)
Neutralizing anti-GPR77 antibody	Deplete CD10+ GPR77+ CAFs	Breast and lung cancer	Inhibit tumor growth	Preclinical	(37)
**Restoring CAFs to a quiescent state**					
Dasatinib	Inhibit PDGFR	Lung cancer	Reduce tumor cells proliferation	Preclinical	(84)
Artemisinin	Suppress TGF-β signaling	Breast cancer	Inhibit cancer cells growth and metastasis	Preclinical	(85)
Ruxolitinib and 5-azacytidine	Restore the fibroblast phenotype of CAFs	Lung and head and neck carcinomas	Reverse invasiveness of CAFs	Preclinical	(86)
GKT137831 [Setanaxib]	Inhibit NOX4	A broad range of cancers	Reverse immune resistance	Preclinical	(87)
Minnelide	Decrease viability of CAFs	Pancreatic cancer	Inhibit tumor growth	Phase l	(88)
Losartan and FOLFIRINOX	Suppress TGF-β signaling	Pancreatic cancer	Reverse tumor immunosuppression	Phase II	(89)
**Blocking the interaction between CAFs and cancer cells**					
LY2109761	Inhibit CTGF and TGF-β signal	Hepatocellular carcinoma	Inhibit tumor growth, intravasation and metastasis	Preclinical	(90)
7E3	Inhibit NRG1 and AKT/MAPK signals	Pancreatic cancer	Inhibit tumor growth and metastasis	Preclinical	(91)
AG490	Inhibit IL-17a and JAK2/STAT3 signaling pathway	Gastric cancer	Inhibit cancer cells growth	Preclinical	(92)
GDC-0449	Inhibit SHH signaling	Pancreatic cancer	Reverse doxorubicin resistance	Preclinical	(93)
RvD1	Inhibit CAFs-derived COMP	Hepatocellular carcinoma	Repress EMT and cancer stemness	Preclinical	(94)
AMD3100 and TN14003	Inhibit CXCR4	HER2 breast cancer	Inhibit cancer cells growth and metastasis	Preclinical	(95)
CAFs-derived WNT2 interference	Restore DC differentiation	Oesophageal squamous cell and colorectal cancer	Enhance immune response	Preclinical	(69)
Ruxolitinib	Suppress JACK/STAT pathway	Pancreatic cancer	Inhibit tumor growth	Phase II	(96)
Nab-paclitaxel and atezolizumab	Disrupt the stroma	Breast cancer	Block pathological collagen accumulation	Phase III	(97)

### CAF-targeted ablation

Targeting CAFs by inhibiting surface markers such as FAP and α-SMA has been extensively explored in pre-clinical studies. Sibrotuzumab, an antibody against FAP, has been tested in phase I clinical trials of colorectal cancer and non-small cell lung carcinoma. In patients with advanced FAP-positive cancer, repeat infusions of sibrotuzumab were safe, but the efficiency in Phase II trials was limited (78). The first clinical inhibitor against FAP activity, Val-boroPro, was used in phase II trials in patients with metastatic colorectal cancer. However, the results were not satisfactory and Val-boroPro had minimal clinical activity (79). In a mouse model, SynCon, a novel FAP DNA vaccine, was able to break tolerance and induce CD8+ and CD4+ immune responses (80). Similarly, the FAP-targeting immunotoxin αFAP-PE38 was used to deplete FAP+ CAFs in a metastatic breast cancer model, thereby decreasing the recruitment of tumor-infiltrating immune cells in the tumor microenvironment and suppressing tumor growth (81). Similar to the depletion of FAP+ CAFs, reduction of α-SMA+ content of stroma through Cellax therapy was confirmed to have effects in inhibiting tumor progression (82). Furthermore, CD10+GPR77+ CAFs were a novel subset that was identified in breast cancer. A neutralizing anti-GPR77 antibody could restore the chemosensitivity of cancer cells (37). Although CAF ablation is effective in some tumor models, the reduction of FAP+ stromal cells are proved to have a relationship with the loss of muscle mass and anemia (83). In addition, CAFs lack specific markers and alter phenotypes at different stage, making targeted therapy difficult. In conclusion, ablation of CAFs in cancer therapy needs cautious consideration, as non-selective removal may have the opposite effect, and the combined application of markers may contribute to more accurate subtype localization.

### Restoring CAFs to a quiescent state

Sustained stimulation of tumor cells will activate some signaling pathways in progenitors, and promote their acquisition of CAF phenotypes and tumor-promoting functions. Strategies to inhibit the expression of some genes in activated CAFs may restore them to a quiescent state, which fails to promote tumor growth and even has tumor-suppressive effects (98). TGF-β and PDGF play crucial roles in the activation of CAFs. Dasatinib, the inhibitor of PDGFR, could reverse the phenotype of CAFs into normal fibroblasts. The proliferation of lung cancer cells was reduced if they were incubated with conditioned medium from CAFs pre-incubated with Dasatinib (84). Similarly, artesunate and dihydroartemisinin from Artemisinin (ART) were shown to suppress TGF-β signaling in CAFs and inhibit tumor growth and metastasis (85). The combination of JAK inhibitor (ruxolitinib) and DNMT inhibitor (5-azacytidine) could restore the fibroblast phenotype and reverse the pro-invasive activity of CAFs in lung cancer and head and neck carcinomas (86). The ROS-producing enzyme NOX4 was upregulated by CAFs in many human cancers, and gene inhibitors convert fibroblasts to CAFs, preventing CAF accumulation and slowing tumor growth (98). Pharmacologic inhibition of NOX4 by GKT137831 [Setanaxib] reversed CAFs to a quiescent state, overcame cancer immune resistance, and improved the prognosis of multiple cancers in a CAF-rich mouse tumor model (87). Minnelide is a water-soluble triptolide prodrug in phase I clinical trials. It is effective in multiple animal models of pancreatic cancer. Minnelide was observed to decrease the viability of CAFs and reduce ECM components such as hyaluronan and collagen, resulting in the suppression of cancer cells (88). Additionally, the use of angiotensin receptor blockers (ARBs) like losartan, converted myofibroblast CAFs to a quiescent state by decreasing the activation of TGF-β, and then alleviated immunosuppression and improved T lymphocyte activity (99). In a phase II clinical trial, the researchers combined losartan with FOLFIRINOX to assess the efficiency of locally advanced pancreatic cancer, and the results showed that the treatment prolonged the prognosis of patients (89).

### Blocking the interaction between CAFs and cancer cells

Compared with depletion of CAFs or reversion of their state, other treatments, such as blocking the interaction between CAFs and cancer cells may be more practical. TGF-β signaling pathway has been proven to be vital in CAF activation and tumor promotion. LY2109761, the TGF-β receptor inhibitor, could suppress tumor growth and metastasis by inhibiting the release of connective tissue growth factor (CTGF) and interrupting the cross-talk between cancer cells and CAFs (90). In preclinical models of pancreatic tumor, neuregulin 1 (NRG1), the ligand of HER3 and HER4 receptors, was secreted by both cancer cells and CAFs. 7E3, as an antibody to NRG1, was demonstrated to prevent tumor growth and metastasis by inhibiting NRG1-mediated HER3 and AKT/MAPK signaling pathways, providing a novel therapeutic option for pancreatic cancer (91). In gastric cancer, IL-17a secreted by CAFs promoted the migration and invasion of cancer cells by activating JAK2/STAT3 signaling pathway. As a neutralizing antibody against IL-17a or JAK2 inhibitors, AG490, could significantly inhibit the effect of CAFs on cancer progression and improve prognosis (92). Furthermore, CAFs in pancreatic cancer were found to interact with tumor cells and hyperactive SHH signaling. A commercial SHH inhibitor, GDC-0449 was reported to reverse fibroblast-induced resistance to doxorubicin in smoothened-positive pancreatic cancer cells. Importantly, the synergistic combination of GDC-0449 with PEG-PCL-Dox exhibited robust antitumor efficiency in a BxPC-3 tumor xenograft model, suggesting a potential strategy for the treatment of fibroblast-enriched pancreatic cancer (93). In hepatocellular carcinoma, the utilize of Resolvin D1 (RvD1) inhibited the paracrine of CAFs-derived cartilage oligomeric matrix protein (COMP) by targeting FPR2/ROS/FOXM1 signaling pathway, and repressed EMT and cancer stemness feature, which might be a potential agent contributing to treatment outcomes (94). The expression of CXCL12 in fibroblasts was considered to be associated with the presence of axillary metastases in HER2 breast cancer, and the suppression of its receptor provided some therapeutic potential. Researchers inhibited CXCR4, the receptor of CXCL12, through the administration of AMD3100 and TN14003, and found the effective suppression of tumor growth and metastasis (95). Similarly, in primary esophageal squamous cell carcinoma and colorectal cancer, WNT2+ CAFs were negatively correlated with active CD8+ T cells. Direct interference with CAF-derived WNT2 could restore DC differentiation and DC-mediated antitumor T-cell response (69). In a phase II clinical trial of pancreatic cancer, ruxolitinib combined with capecitabine was used in patients with metastatic pancreatic cancer who had failed to respond to gemcitabine. The results showed that patients treated with ruxolitinib had longer overall survival and better prognosis, supporting the potential clinical benefit of JAK1/JAK2 inhibitor ruxolitinib (96). Additionally, the stromal-disrupting effect of Nab-paclitaxel was reported in pancreatic cancer therapy (100). In a phase III clinical trial, nab-paclitaxel combined with atezolizumab was tested in patients with unrespectable, locally advanced or metastatic triple-negative breast cancer and showed longer overall survival (97).

## Conclusions

Since the concept of CAFs was proposed in the early 1990s, CAFs have attracted extensive attention in cancer biology. Previous studies have led to a better understanding of the heterogeneity of CAF origins, phenotypes and functions. CAFs are the main cell types in tumor microenvironment which affect the occurrence, and development of cancer cells. They have rich cellular sources and precursor cells such as normal fibroblasts and mesenchymal stem cells have been shown to be the major sources. CAFs are not a cell type but heterogeneous functional subpopulations. Based on the surface markers, CAFs are divided into several subtypes, which have different biological functions. CAF subtypes identified in different cancer types may play opposite roles in cancer progression, such as tumor-promoting and tumor-suppressive functions. CAFs have great potential in clinical applications. Several preclinical studies and ongoing clinical trials have shown that strategies targeting CAFs are possible in cancer therapy. However, there are still some challenges in translating CAF research into clinical benefit. First, the concrete origins of CAFs in specific cancer types remains elusive. In addition, most studies on the origin of CAFs have been performed *in vitro* and lack appropriate clinical validation. The use of lineage tracing methods will greatly solve these problems in future studies. Second, the lack of uniform nomenclature for CAF subpopulations in different cancer types makes it difficult to compare CAF subgroups in distinct tumors. It would be useful to name them by combining analysis of cell lineage, surface markers, functions and clinical relevance. Additionally, there is still a lack of curate classification of CAF subtypes. Advanced strategies, such as single-cell RNA sequencing, mass spectrometry-based time-of-flight flow cytometry (CyTOF), multiple flow cytometry and multiple immunostaining, may be helpful to accurately classify CAF subtypes. Finally, although many experiments targeting CAFs to improve cancer therapy have been conducted in preclinical models and clinical trials, most of them have failed to pass phase II clinical trials. It has not yet reached practical application. To overcome this limitation, more detailed experimental designs and more clinical samples are needed, and the combination of these CAF-targeting approaches with existing therapies may be beneficial. Overall, it is critical to accurately understand the underlying mechanisms of action between CAFs and tumor cells. It is also important to understand CAF-targeting therapies at the molecular, cellular, and systemic levels based on the interactions between CAFs and tumor cells, to find the most appropriate strategies and avoid adverse effects. In addition, tracing the origins of CAFs may be a key factor in achieving the clinical application of CAF-targeting strategies and avoiding side effects. With the resolution of these problems, CAF-derived therapies are expected to provide new support for clinical cancer therapy in the near future.

## Author contributions

CW wrote the manuscript and designed the figures. JG, HG, and XXZ assisted in the manuscript writing and figures drawing. XZ and RJ revised the manuscript. All authors contributed to the article and approved the submitted version.

## Funding

The present study was supported by Distinguished Young Scholar Project of Jiangsu Natural Science Foundation (BK20200043), the National Natural Science Foundation of China (Grant no. 81702429, 81672416, 81972310), Natural Science Foundation of the Jiangsu Province (Grant No. BK20170561), Zhenjiang Science & Technology Program (Grant No. SH2019051).

## Acknowledgments

The authors would like to thank literature support from Jiangsu University library and the support from fundings.

## Conflict of interest

The authors declare that the research was conducted in the absence of any commercial or financial relationships that could be construed as a potential conflict of interest.

## Publisher’s note

All claims expressed in this article are solely those of the authors and do not necessarily represent those of their affiliated organizations, or those of the publisher, the editors and the reviewers. Any product that may be evaluated in this article, or claim that may be made by its manufacturer, is not guaranteed or endorsed by the publisher.

## References

[1] Chen Y McAndrews KM Kalluri R. Clinical and therapeutic relevance of cancer-associated fibroblasts. Nat Rev Clin Oncol (2021) 18:792–804. doi: 10.1038/s41571-021-00546-5 34489603 PMC8791784

[2] Sahai E Astsaturov I Cukierman E DeNardo DG Egeblad M Evans RM. A framework for advancing our understanding of cancer-associated fibroblasts. Nat Rev Cancer (2020) 20:174–86. doi: 10.1038/s41568-019-0238-1 PMC7046529 31980749

[3] Liao Z Tan ZW Zhu P Tan NS. Cancer-associated fibroblasts in tumor microenvironment - accomplices in tumor malignancy. Cell Immunol (2019) 343:103729. doi: 10.1016/j.cellimm.2017.12.003 29397066

[4] von Ahrens D Bhagat TD Nagrath D Maitra A Verma A. The role of stromal cancer-associated fibroblasts in pancreatic cancer. J Hematol Oncol (2017) 10:76. doi: 10.1186/s13045-017-0448-5 28351381 PMC5371211

[5] Miyai Y Esaki N Takahashi M Enomoto A. Cancer-associated fibroblasts that restrain cancer progression: Hypotheses and perspectives. Cancer Sci (2020) 111:1047–57. doi: 10.1111/cas.14346 PMC7156845 32060987

[6] Driskell RR Lichtenberger BM Hoste E Kretzschmar K Simons BD Charalambous M. Distinct fibroblast lineages determine dermal architecture in skin development and repair. Nature (2013) 504:277–81. doi: 10.1038/nature12783 PMC3868929 24336287

[7] Dulauroy S Di Carlo SE Langa F Eberl G Peduto L. Lineage tracing and genetic ablation of ADAM12(+) perivascular cells identify a major source of profibrotic cells during acute tissue injury. Nat Med (2012) 18:1262–70. doi: 10.1038/nm.2848 22842476

[8] Ringuette Goulet C Bernard G Tremblay S Chabaud S Bolduc S Pouliot F. Exosomes induce fibroblast differentiation into cancer-associated fibroblasts through TGFβ signaling. Mol Cancer Res (2018) 16:1196–204. doi: 10.1158/1541-7786.Mcr-17-0784 29636362

[9] Karnoub AE Dash AB Vo AP Sullivan A Brooks MW Bell GW. Mesenchymal stem cells within tumour stroma promote breast cancer metastasis. Nature (2007) 449:557–63. doi: 10.1038/nature06188 17914389

[10] Öhlund D Handly-Santana A Biffi G Elyada E Almeida AS Ponz-Sarvise M. Distinct populations of inflammatory fibroblasts and myofibroblasts in pancreatic cancer. J Exp Med (2017) 214:579–96. doi: 10.1084/jem.20162024 PMC5339682 28232471

[11] Suzuki M Ramezanpour M Cooksley C Li J Nakamaru Y Homma A. Sirtuin-1 controls poly (I:C)-dependent matrix metalloproteinase 9 activation in primary human nasal epithelial cells. Am J Respir Cell Mol Biol (2018) 59:500–10. doi: 10.1165/rcmb.2017-0415OC 29767533

[12] Zeisberg EM Potenta S Xie L Zeisberg M Kalluri R. Discovery of endothelial to mesenchymal transition as a source for carcinoma-associated fibroblasts. Cancer Res (2007) 67:10123–8. doi: 10.1158/0008-5472.Can-07-3127 17974953

[13] Iyoshi S Yoshihara M Nakamura K Sugiyama M Koya Y Kitami K. Pro-tumoral behavior of omental adipocyte-derived fibroblasts in tumor microenvironment at the metastatic site of ovarian cancer. Int J Cancer (2021) 149:1961–72. doi: 10.1002/ijc.33770 34469585

[14] Ning X Zhang H Wang C Song X. Exosomes released by gastric cancer cells induce transition of pericytes into cancer-associated fibroblasts. Med Sci Monitor (2018) 24:2350–9. doi: 10.12659/msm.906641 PMC5922989 29668670

[15] Nair N Calle AS Zahra MH Prieto-Vila M Oo AKK Hurley L. A cancer stem cell model as the point of origin of cancer-associated fibroblasts in tumor microenvironment. Sci Rep (2017) 7:6838. doi: 10.1038/s41598-017-07144-5 28754894 PMC5533745

[16] McDonald LT Russell DL Kelly RR Xiong Y Motamarry A Patel RK. Hematopoietic stem cell-derived cancer-associated fibroblasts are novel contributors to the pro-tumorigenic microenvironment. Neoplasia (New York NY) (2015) 17:434–48. doi: 10.1016/j.neo.2015.04.004 PMC4468366 26025666

[17] Bhowmick NA Chytil A Plieth D Gorska AE Dumont N Shappell S. TGF-beta signaling in fibroblasts modulates the oncogenic potential of adjacent epithelia. Science (2004) 303:848–51. doi: 10.1126/science.1090922 14764882

[18] Cadamuro M Brivio S Mertens J Vismara M Moncsek A Milani C. Platelet-derived growth factor-d enables liver myofibroblasts to promote tumor lymphangiogenesis in cholangiocarcinoma. J Hepatol (2019) 70:700–9. doi: 10.1016/j.jhep.2018.12.004 PMC10878126 30553841

[19] Fang T Lv H Lv G Li T Wang C Han Q. Tumor-derived exosomal miR-1247-3p induces cancer-associated fibroblast activation to foster lung metastasis of liver cancer. Nat Commun (2018) 9:191. doi: 10.1038/s41467-017-02583-0 29335551 PMC5768693

[20] Xue B Chuang CH Prosser HM Fuziwara CS Chan C Sahasrabudhe N. miR-200 deficiency promotes lung cancer metastasis by activating notch signaling in cancer-associated fibroblasts. Genes Dev (2021) 35:1109–22. doi: 10.1101/gad.347344.120 PMC8336896 34301766

[21] Zou B Liu X Zhang B Gong Y Cai C Li P. The expression of FAP in hepatocellular carcinoma cells is induced by hypoxia and correlates with poor clinical outcomes. J Cancer (2018) 9:3278–86. doi: 10.7150/jca.25775 PMC6160687 30271487

[22] Barcellos-de-Souza P Comito G Pons-Segura C Taddei ML Gori V Becherucci V. Mesenchymal stem cells are recruited and activated into carcinoma-associated fibroblasts by prostate cancer microenvironment-derived TGF-β1. Stem Cells (Dayton Ohio) (2016) 34:2536–47. doi: 10.1002/stem.2412 27300750

[23] Tan HX Xiao ZG Huang T Fang ZX Liu Y Huang ZC. CXCR4/TGF-β1 mediated self-differentiation of human mesenchymal stem cells to carcinoma-associated fibroblasts and promoted colorectal carcinoma development. Cancer Biol Ther (2020) 21:248–57. doi: 10.1080/15384047.2019.1685156 PMC7012141 31818187

[24] Zhao L Ji G Le X Luo Z Wang C Feng M. An integrated analysis identifies STAT4 as a key regulator of ovarian cancer metastasis. Oncogene (2017) 36:3384–96. doi: 10.1038/onc.2016.487 28114283

[25] Zhu H Guo S Zhang Y Yin J Yin W Tao S. Proton-sensing GPCR-YAP signalling promotes cancer-associated fibroblast activation of mesenchymal stem cells. Int J Biol Sci (2016) 12:389–96. doi: 10.7150/ijbs.13688 PMC4807159 27019624

[26] Biffi G Oni TE Spielman B Hao Y Elyada E Park Y. IL1-induced JAK/STAT signaling is antagonized by TGFβ to shape CAF heterogeneity in pancreatic ductal adenocarcinoma. Cancer Discov (2019) 9:282–301. doi: 10.1158/2159-8290.Cd-18-0710 30366930 PMC6368881

[27] Kanzaki R Pietras K. Heterogeneity of cancer-associated fibroblasts: Opportunities for precision medicine. Cancer Sci (2020) 111:2708–17. doi: 10.1111/cas.14537 PMC7419037 32573845

[28] Nomura S. Identification, friend or foe: Vimentin and α-smooth muscle actin in cancer-associated fibroblasts. Ann Surg Oncol (2019) 26:4191–2. doi: 10.1245/s10434-019-07894-8 31605319

[29] Yamao T Yamashita YI Yamamura K Nakao Y Tsukamoto M Nakagawa S. Cellular senescence, represented by expression of caveolin-1, in cancer-associated fibroblasts promotes tumor invasion in pancreatic cancer. Ann Surg Oncol (2019) 26:1552–9. doi: 10.1245/s10434-019-07266-2 30805811

[30] Shimizu K Kirita K Aokage K Kojima M Hishida T Kuwata T. Clinicopathological significance of caveolin-1 expression by cancer-associated fibroblasts in lung adenocarcinoma. J Cancer Res Clin Oncol (2017) 143:321–8. doi: 10.1007/s00432-016-2285-2 PMC11819325 27771795

[31] Kobayashi H Gieniec KA Lannagan TRM Wang T Asai N Mizutani Y. The origin and contribution of cancer-associated fibroblasts in colorectal carcinogenesis. Gastroenterology (2022) 162:890–906. doi: 10.1053/j.gastro.2021.11.037 34883119 PMC8881386

[32] Zaghdoudi S Decaup E Belhabib I Samain R Cassant-Sourdy S Rochotte J. FAK activity in cancer-associated fibroblasts is a prognostic marker and a druggable key metastatic player in pancreatic cancer. EMBO Mol Med (2020) 12:e12010. doi: 10.15252/emmm.202012010 33025708 PMC7645544

[33] Elyada E Bolisetty M Laise P Flynn WF Courtois ET Burkhart RA. Cross-species single-cell analysis of pancreatic ductal adenocarcinoma reveals antigen-presenting cancer-associated fibroblasts. Cancer Discov (2019) 9:1102–23. doi: 10.1158/2159-8290.Cd-19-0094 PMC6727976 31197017

[34] Neuzillet C Tijeras-Raballand A Ragulan C Cros J Patil Y Martinet M. Inter- and intra-tumoural heterogeneity in cancer-associated fibroblasts of human pancreatic ductal adenocarcinoma. J Pathol (2019) 248:51–65. doi: 10.1002/path.5224 30575030 PMC6492001

[35] Wang Y Liang Y Xu H Zhang X Mao T Cui J. Single-cell analysis of pancreatic ductal adenocarcinoma identifies a novel fibroblast subtype associated with poor prognosis but better immunotherapy response. Cell Discov (2021) 7:36. doi: 10.1038/s41421-021-00271-4 34035226 PMC8149399

[36] Pelon F Bourachot B Kieffer Y Magagna I Mermet-Meillon F Bonnet I. Cancer-associated fibroblast heterogeneity in axillary lymph nodes drives metastases in breast cancer through complementary mechanisms. Nat Commun (2020) 11:404. doi: 10.1038/s41467-019-14134-w 31964880 PMC6972713

[37] Su S Chen J Yao H Liu J Yu S Lao L. CD10(+)GPR77(+) cancer-associated fibroblasts promote cancer formation and chemoresistance by sustaining cancer stemness. Cell (2018) 172:841–56.e16. doi: 10.1016/j.cell.2018.01.009 29395328

[38] Bartoschek M Oskolkov N Bocci M Lövrot J Larsson C Sommarin M. Spatially and functionally distinct subclasses of breast cancer-associated fibroblasts revealed by single cell RNA sequencing. Nat Commun (2018) 9:5150. doi: 10.1038/s41467-018-07582-3 30514914 PMC6279758

[39] Patel AK Vipparthi K Thatikonda V Arun I Bhattacharjee S Sharan R. A subtype of cancer-associated fibroblasts with lower expression of alpha-smooth muscle actin suppresses stemness through BMP4 in oral carcinoma. Oncogenesis (2018) 7:78. doi: 10.1038/s41389-018-0087-x 30287850 PMC6172238

[40] Li X Sun Z Peng G Xiao Y Guo J Wu B. Single-cell RNA sequencing reveals a pro-invasive cancer-associated fibroblast subgroup associated with poor clinical outcomes in patients with gastric cancer. Theranostics (2022) 12:620–38. doi: 10.7150/thno.60540 PMC8692898 34976204

[41] Li H Courtois ET Sengupta D Tan Y Chen KH Goh JJL. Reference component analysis of single-cell transcriptomes elucidates cellular heterogeneity in human colorectal tumors. Nat Genet (2017) 49:708–18. doi: 10.1038/ng.3818 28319088

[42] Hu H Piotrowska Z Hare PJ Chen H Mulvey HE Mayfield A. Three subtypes of lung cancer fibroblasts define distinct therapeutic paradigms. Cancer Cell (2021) 39:1531–47.e10. doi: 10.1016/j.ccell.2021.09.003 34624218 PMC8578451

[43] Yin Z Dong C Jiang K Xu Z Li R Guo K. Heterogeneity of cancer-associated fibroblasts and roles in the progression, prognosis, and therapy of hepatocellular carcinoma. J Hematol Oncol (2019) 12:101. doi: 10.1186/s13045-019-0782-x 31547836 PMC6757399

[44] Hussain A Voisin V Poon S Karamboulas C Bui NHB Meens J. Distinct fibroblast functional states drive clinical outcomes in ovarian cancer and are regulated by TCF21. J Exp Med (2020) 217:e20191094. doi: 10.1084/jem.20191094 32434219 PMC7398174

[45] Chen X Song E. Turning foes to friends: targeting cancer-associated fibroblasts. Nat Rev Drug Discov (2019) 18:99–115. doi: 10.1038/s41573-018-0004-1 30470818

[46] Papalexi E Satija R. Single-cell RNA sequencing to explore immune cell heterogeneity. Nat Rev Immunol (2018) 18:35–45. doi: 10.1038/nri.2017.76 28787399

[47] Zhang Q Lou Y Yang J Wang J Feng J Zhao Y. Integrated multiomic analysis reveals comprehensive tumour heterogeneity and novel immunophenotypic classification in hepatocellular carcinomas. Gut (2019) 68:2019–31. doi: 10.1136/gutjnl-2019-318912 PMC6839802 31227589

[48] Ker HG Coura-Vital W Valadares DG Aguiar-Soares RDO de Brito RCF Veras PST. Multiplex flow cytometry serology to diagnosis of canine visceral leishmaniasis. Appl Microbiol Biotechnol (2019) 103:8179–90. doi: 10.1007/s00253-019-10068-x 31388731

[49] Dong LQ Peng LH Ma LJ Liu DB Zhang S Luo SZ. Heterogeneous immunogenomic features and distinct escape mechanisms in multifocal hepatocellular carcinoma. J Hepatol (2020) 72:896–908. doi: 10.1016/j.jhep.2019.12.014 31887370

[50] Chen Z Zhou L Liu L Hou Y Xiong M Yang Y. Single-cell RNA sequencing highlights the role of inflammatory cancer-associated fibroblasts in bladder urothelial carcinoma. Nat Commun (2020) 11:5077. doi: 10.1038/s41467-020-18916-5 33033240 PMC7545162

[51] Wang Y Lan W Xu M Song J Mao J Li C. Cancer-associated fibroblast-derived SDF-1 induces epithelial-mesenchymal transition of lung adenocarcinoma *via* CXCR4/β-catenin/PPARδ signalling. Cell Death Dis (2021) 12:214. doi: 10.1038/s41419-021-03509-x 33637678 PMC7910618

[52] Wen S Hou Y Fu L Xi L Yang D Zhao M. Cancer-associated fibroblast (CAF)-derived IL32 promotes breast cancer cell invasion and metastasis *via* integrin β3-p38 MAPK signalling. Cancer Lett (2019) 442:320–32. doi: 10.1016/j.canlet.2018.10.015 30391782

[53] Ren J Smid M Iaria J Salvatori DCF van Dam H Zhu HJ. Cancer-associated fibroblast-derived gremlin 1 promotes breast cancer progression. Breast Cancer Res (2019) 21:109. doi: 10.1186/s13058-019-1194-0 31533776 PMC6751614

[54] Wang R Sun Y Yu W Yan Y Qiao M Jiang R. Downregulation of miRNA-214 in cancer-associated fibroblasts contributes to migration and invasion of gastric cancer cells through targeting FGF9 and inducing EMT. J Exp Clin Cancer Res (2019) 38:20. doi: 10.1186/s13046-018-0995-9 30646925 PMC6334467

[55] Ji Q Zhou L Sui H Yang L Wu X Song Q. Primary tumors release ITGBL1-rich extracellular vesicles to promote distal metastatic tumor growth through fibroblast-niche formation. Nat Commun (2020) 11:1211. doi: 10.1038/s41467-020-14869-x 32139701 PMC7058049

[56] Hu JL Wang W Lan XL Zeng ZC Liang YS Yan YR. CAFs secreted exosomes promote metastasis and chemotherapy resistance by enhancing cell stemness and epithelial-mesenchymal transition in colorectal cancer. Mol Cancer (2019) 18:91. doi: 10.1186/s12943-019-1019-x 31064356 PMC6503554

[57] Gascard P Tlsty TD. Carcinoma-associated fibroblasts: Orchestrating the composition of malignancy. Genes Dev (2016) 30:1002–19. doi: 10.1101/gad.279737.116 PMC4863733 27151975

[58] Tao L Huang G Wang R Pan Y He Z Chu X. Cancer-associated fibroblasts treated with cisplatin facilitates chemoresistance of lung adenocarcinoma through IL-11/IL-11R/STAT3 signaling pathway. Sci Rep (2016) 6:38408. doi: 10.1038/srep38408 27922075 PMC5138853

[59] Gao Y Li X Zeng C Liu C Hao Q Li W. CD63(+) cancer-associated fibroblasts confer tamoxifen resistance to breast cancer cells through exosomal miR-22. Advanced Sci (Weinheim Baden-Wurttemberg Germany) (2020) 7:2002518. doi: 10.1002/advs.202002518 PMC7610308 33173749

[60] Zhang H Deng T Liu R Ning T Yang H Liu D. CAF secreted miR-522 suppresses ferroptosis and promotes acquired chemo-resistance in gastric cancer. Mol Cancer (2020) 19:43. doi: 10.1186/s12943-020-01168-8 32106859 PMC7045485

[61] Zhai J Shen J Xie G Wu J He M Gao L. Cancer-associated fibroblasts-derived IL-8 mediates resistance to cisplatin in human gastric cancer. Cancer Lett (2019) 454:37–43. doi: 10.1016/j.canlet.2019.04.002 30978440

[62] Wei L Ye H Li G Lu Y Zhou Q Zheng S. Cancer-associated fibroblasts promote progression and gemcitabine resistance *via* the SDF-1/SATB-1 pathway in pancreatic cancer. Cell Death Dis (2018) 9:1065. doi: 10.1038/s41419-018-1104-x 30337520 PMC6194073

[63] Richards KE Zeleniak AE Fishel ML Wu J Littlepage LE Hill R. Cancer-associated fibroblast exosomes regulate survival and proliferation of pancreatic cancer cells. Oncogene (2017) 36:1770–8. doi: 10.1038/onc.2016.353 PMC5366272 27669441

[64] Chen PY Wei WF Wu HZ Fan LS Wang W. Cancer-associated fibroblast heterogeneity: A factor that cannot be ignored in immune microenvironment remodeling. Front Immunol (2021) 12:2021.671595. doi: 10.3389/fimmu.2021.671595 PMC8297463 34305902

[65] Costa A Kieffer Y Scholer-Dahirel A Pelon F Bourachot B Cardon M. Fibroblast heterogeneity and immunosuppressive environment in human breast cancer. Cancer Cell (2018) 33:463–79.e10. doi: 10.1016/j.ccell.2018.01.011 29455927

[66] Zhao X Ding L Lu Z Huang X Jing Y Yang Y. Diminished CD68(+) cancer-associated fibroblast subset induces regulatory T-cell (Treg) infiltration and predicts poor prognosis of oral squamous cell carcinoma patients. Am J Pathol (2020) 190:886–99. doi: 10.1016/j.ajpath.2019.12.007 32035062

[67] Dou D Ren X Han M Xu X Ge X Gu Y. Cancer-associated fibroblasts-derived exosomes suppress immune cell function in breast cancer *via* the miR-92/PD-L1 pathway. Front Immunol (2020) 11:2020.02026. doi: 10.3389/fimmu.2020.02026 33162971 PMC7581790

[68] Li Z Zhou J Zhang J Li S Wang H Du J. Cancer-associated fibroblasts promote PD-L1 expression in mice cancer cells *via* secreting CXCL5. Int J Cancer (2019) 145:1946–57. doi: 10.1002/ijc.32278 PMC6767568 30873585

[69] Huang TX Tan XY Huang HS Li YT Liu BL Liu KS. Targeting cancer-associated fibroblast-secreted WNT2 restores dendritic cell-mediated antitumour immunity. Gut (2022) 71:333–44. doi: 10.1136/gutjnl-2020-322924 PMC8762012 33692094

[70] Cheng JT Deng YN Yi HM Wang GY Fu BS Chen WJ. Hepatic carcinoma-associated fibroblasts induce IDO-producing regulatory dendritic cells through IL-6-mediated STAT3 activation. Oncogenesis (2016) 5:e198. doi: 10.1038/oncsis.2016.7 26900950 PMC5154347

[71] Cheng Y Li H Deng Y Tai Y Zeng K Zhang Y. Cancer-associated fibroblasts induce PDL1+ neutrophils through the IL6-STAT3 pathway that foster immune suppression in hepatocellular carcinoma. Cell Death Dis (2018) 9:422. doi: 10.1038/s41419-018-0458-4 29556041 PMC5859264

[72] Shani O Vorobyov T Monteran L Lavie D Cohen N Raz Y. Fibroblast-derived IL33 facilitates breast cancer metastasis by modifying the immune microenvironment and driving type 2 immunity. Cancer Res (2020) 80:5317–29. doi: 10.1158/0008-5472.Can-20-2116 PMC7611300 33023944

[73] Özdemir BC Pentcheva-Hoang T Carstens JL Zheng X Wu CC Simpson TR. Depletion of carcinoma-associated fibroblasts and fibrosis induces immunosuppression and accelerates pancreas cancer with reduced survival. Cancer Cell (2014) 25:719–34. doi: 10.1016/j.ccr.2014.04.005 PMC4180632 24856586

[74] Rhim AD Oberstein PE Thomas DH Mirek ET Palermo CF Sastra SA. Stromal elements act to restrain, rather than support, pancreatic ductal adenocarcinoma. Cancer Cell (2014) 25:735–47. doi: 10.1016/j.ccr.2014.04.021 PMC4096698 24856585

[75] Brechbuhl HM Finlay-Schultz J Yamamoto TM Gillen AE Cittelly DM Tan AC. Fibroblast subtypes regulate responsiveness of luminal breast cancer to estrogen. Clin Cancer Res (2017) 23:1710–21. doi: 10.1158/1078-0432.Ccr-15-2851 PMC5378660 27702820

[76] Mizutani Y Kobayashi H Iida T Asai N Masamune A Hara A. Meflin-positive cancer-associated fibroblasts inhibit pancreatic carcinogenesis. Cancer Res (2019) 79:5367–81. doi: 10.1158/0008-5472.Can-19-0454 31439548

[77] Fiori ME Di Franco S Villanova L Bianca P Stassi G De Maria R. Cancer-associated fibroblasts as abettors of tumor progression at the crossroads of EMT and therapy resistance. Mol Cancer (2019) 18:70. doi: 10.1186/s12943-019-0994-2 30927908 PMC6441236

[78] Scott AM Wiseman G Welt S Adjei A Lee FT Hopkins W. A phase I dose-escalation study of sibrotuzumab in patients with advanced or metastatic fibroblast activation protein-positive cancer. Clin Cancer Res (2003) 9:1639–47. 12738716

[79] Narra K Mullins SR Lee HO Strzemkowski-Brun B Magalong K Christiansen VJ. Phase II trial of single agent Val-boroPro (Talabostat) inhibiting fibroblast activation protein in patients with metastatic colorectal cancer. Cancer Biol Ther (2007) 6:1691–9. doi: 10.4161/cbt.6.11.4874 18032930

[80] Duperret EK Trautz A Ammons D Perales-Puchalt A Wise MC Yan J. Alteration of the tumor stroma using a consensus DNA vaccine targeting fibroblast activation protein (FAP) synergizes with antitumor vaccine therapy in mice. Clin Cancer Res (2018) 24:1190–201. doi: 10.1158/1078-0432.Ccr-17-2033 PMC5844837 29269377

[81] Fang J Xiao L Joo KI Liu Y Zhang C Liu S. A potent immunotoxin targeting fibroblast activation protein for treatment of breast cancer in mice. Int J Cancer (2016) 138:1013–23. doi: 10.1002/ijc.29831 PMC4715643 26334777

[82] Murakami M Ernsting MJ Undzys E Holwell N Foltz WD Li SD. Docetaxel conjugate nanoparticles that target α-smooth muscle actin-expressing stromal cells suppress breast cancer metastasis. Cancer Res (2013) 73:4862–71. doi: 10.1158/0008-5472.Can-13-0062 23907638

[83] Haubeiss S Schmid JO Mürdter TE Sonnenberg M Friedel G van der Kuip H. Dasatinib reverses cancer-associated fibroblasts (CAFs) from primary lung carcinomas to a phenotype comparable to that of normal fibroblasts. Mol Cancer (2010) 9:168. doi: 10.1186/1476-4598-9-168 20579391 PMC2907332

[84] Yao Y Guo Q Cao Y Qiu Y Tan R Yu Z. Artemisinin derivatives inactivate cancer-associated fibroblasts through suppressing TGF-β signaling in breast cancer. J Exp Clin Cancer Res (2018) 37:282. doi: 10.1186/s13046-018-0960-7 30477536 PMC6258160

[85] Albrengues J Bertero T Grasset E Bonan S Maiel M Bourget I. Epigenetic switch drives the conversion of fibroblasts into proinvasive cancer-associated fibroblasts. Nat Commun (2015) 6:10204. doi: 10.1038/ncomms10204 26667266 PMC4682161

[86] Ford K Hanley CJ Mellone M Szyndralewiez C Heitz F Wiesel P. NOX4 inhibition potentiates immunotherapy by overcoming cancer-associated fibroblast-mediated CD8 T-cell exclusion from tumors. Cancer Res (2020) 80:1846–60. doi: 10.1158/0008-5472.Can-19-3158 PMC7611230 32122909

[87] Banerjee S Modi S McGinn O Zhao X Dudeja V Ramakrishnan S. Impaired synthesis of stromal components in response to minnelide improves vascular function, drug delivery, and survival in pancreatic cancer. Clin Cancer Res (2016) 22:415–25. doi: 10.1158/1078-0432.Ccr-15-1155 PMC4716007 26405195

[88] Murphy JE Wo JY Ryan DP Clark JW Jiang W Yeap BY. Total neoadjuvant therapy with FOLFIRINOX in combination with losartan followed by chemoradiotherapy for locally advanced pancreatic cancer: A phase 2 clinical trial. JAMA Oncol (2019) 5:1020–7. doi: 10.1001/jamaoncol.2019.0892 PMC6547247 31145418

[89] Mazzocca A Fransvea E Dituri F Lupo L Antonaci S Giannelli G. Down-regulation of connective tissue growth factor by inhibition of transforming growth factor beta blocks the tumor-stroma cross-talk and tumor progression in hepatocellular carcinoma. Hepatol (Baltimore Md) (2010) 51:523–34. doi: 10.1002/hep.23285 19821534

[90] Ogier C Colombo PE Bousquet C Canterel-Thouennon L Sicard P Garambois V. Targeting the NRG1/HER3 pathway in tumor cells and cancer-associated fibroblasts with an anti-neuregulin 1 antibody inhibits tumor growth in pre-clinical models of pancreatic cancer. Cancer Lett (2018) 432:227–36. doi: 10.1016/j.canlet.2018.06.023 29935372

[91] Zhang J Li S Zhao Y Ma P Cao Y Liu C. Cancer-associated fibroblasts promote the migration and invasion of gastric cancer cells *via* activating IL-17a/JAK2/STAT3 signaling. Ann Trans Med (2020) 8:877. doi: 10.21037/atm-20-4843 PMC7396760 32793721

[92] Zhou Q Zhou Y Liu X Shen Y. GDC-0449 improves the antitumor activity of nano-doxorubicin in pancreatic cancer in a fibroblast-enriched microenvironment. Sci Rep (2017) 7:13379. doi: 10.1038/s41598-017-13869-0 29042665 PMC5645386

[93] Sun L Wang Y Wang L Yao B Chen T Li Q. Resolvin D1 prevents epithelial-mesenchymal transition and reduces the stemness features of hepatocellular carcinoma by inhibiting paracrine of cancer-associated fibroblast-derived COMP. J Exp Clin Cancer Res (2019) 38:170. doi: 10.1186/s13046-019-1163-6 30999932 PMC6472102

[94] Lefort S Thuleau A Kieffer Y Sirven P Bieche I Marangoni E. CXCR4 inhibitors could benefit to HER2 but not to triple-negative breast cancer patients. Oncogene (2017) 36:1211–22. doi: 10.1038/onc.2016.284 PMC5340801 27669438

[95] Hurwitz HI Uppal N Wagner SA Bendell JC Beck JT Wade SM 3rd. Randomized, double-blind, phase II study of ruxolitinib or placebo in combination with capecitabine in patients with metastatic pancreatic cancer for whom therapy with gemcitabine has failed. J Clin Oncol (2015) 33:4039–47. doi: 10.1200/jco.2015.61.4578 PMC5089161 26351344

[96] Schmid P Rugo HS Adams S Schneeweiss A Barrios CH Iwata H. Atezolizumab plus nab-paclitaxel as first-line treatment for unresectable, locally advanced or metastatic triple-negative breast cancer (IMpassion130): updated efficacy results from a randomised, double-blind, placebo-controlled, phase 3 trial. Lancet Oncol (2020) 21:44–59. doi: 10.1016/s1470-2045(19)30689-8 31786121

[97] Roberts EW Deonarine A Jones JO Denton AE Feig C Lyons SK. Depletion of stromal cells expressing fibroblast activation protein-α from skeletal muscle and bone marrow results in cachexia and anemia. J Exp Med (2013) 210:1137–51. doi: 10.1084/jem.20122344 PMC3674708 23712428

[98] Hanley CJ Mellone M Ford K Thirdborough SM Mellows T Frampton SJ. Targeting the myofibroblastic cancer-associated fibroblast phenotype through inhibition of NOX4. J Natl Cancer Institute (2018) 110:109–20. doi: 10.1093/jnci/djx121 PMC5903651 28922779

[99] Chauhan VP Chen IX Tong R Ng MR Martin JD Naxerova K. Reprogramming the microenvironment with tumor-selective angiotensin blockers enhances cancer immunotherapy. Proc Natl Acad Sci U.S.A. (2019) 116:10674–80. doi: 10.1073/pnas.1819889116 PMC6561160 31040208

[100] Feng R Morine Y Ikemoto T Imura S Iwahashi S Saito Y. Nab-paclitaxel interrupts cancer-stromal interaction through c-X-C motif chemokine 10-mediated interleukin-6 downregulation *in vitro* . Cancer Sci (2018) 109:2509–19. doi: 10.1111/cas.13694 PMC6113502 29902349

